# Characterizing semen parameters and their association with reactive oxygen species in infertile men

**DOI:** 10.1186/1477-7827-12-33

**Published:** 2014-05-07

**Authors:** Ashok Agarwal, Rakesh K Sharma, Reecha Sharma, Mourad Assidi, Adel M Abuzenadah, Saad Alshahrani, Damayanthi Durairajanayagam, Edmund Sabanegh

**Affiliations:** 1 Center for Reproductive Medicine, Cleveland Clinic, Cleveland, Ohio 44106, USA; 2 Health Services Department, Saint Joseph University, 5600 City Avenue, Philadelphia, PA 19131, USA; 3 Center of Excellence in Genomic Medicine Research, King AbdulAziz University, Jeddah, Saudi Arabia; 4 KACST Technology Innovation Center in Personalized Medicine at King AbdulAziz University, Jeddah, Saudi Arabia; 5 Department of Surgery, Salman Bin Abdulaziz University, College of Medicine, Alkharj, Saudi Arabia; 6 MARA University of Technology, Sungai Buloh Campus, Jalan Hospital, 47000 Sungai Buloh, Selangor Darul Ehsan, Malaysia

**Keywords:** Sperm, Motility, Sperm quality, Reactive oxygen species, Infertility

## Abstract

**Background:**

A routine semen analysis is a first step in the laboratory evaluation of the infertile male. In addition, other tests such as measurement of reactive oxygen species can provide additional information regarding the etiology of male infertility. The objective of this study was to investigate the association of semen parameters with reactive oxygen species (ROS) in two groups: healthy donors of unproven and proven fertility and infertile men. In addition, we sought to establish an ROS cutoff value in seminal plasma at which a patient may be predicted to be infertile.

**Methods:**

Seminal ejaculates from 318 infertile patients and 56 donors, including those with proven fertility were examined for semen parameters and ROS levels. Correlations were determined between traditional semen parameters and levels of ROS among the study participants. ROS levels were measured using chemiluminescence assay. Receiver operating characteristic curves were obtained to calculate a cutoff value for these tests.

**Results:**

Proven Donors (n = 28) and Proven Donors within the past 2 years (n = 16) showed significantly better semen parameters than All Patients group (n = 318). Significantly lower ROS levels were seen in the two Proven Donor groups compared with All Patients. The cutoff value of ROS in Proven Donors was determined to be 91.9 RLU/s with a specificity of 68.8% and a sensitivity of 93.8%.

**Conclusions:**

Infertile men, irrespective of their clinical diagnoses, have reduced semen parameters and elevated ROS levels compared to proven fertile men who have established a pregnancy recently or in the past. Reactive oxygen species are negatively correlated with traditional semen parameters such as concentration, motility and morphology. Measuring ROS levels in the seminal ejaculates provides clinically-relevant information to clinicians.

## Background

Infertility affects 15% of couples, with male factor dysfunction contributing to 50% of all cases [1]. The common causes of male infertility include varicocele, genital tract infections, radiation, chemotherapy, erectile dysfunction, gene mutations and aneuploidy [2]. Among all cases of male infertility, 40-50% are characterized as ‘idiopathic’ [3].

One of the main causes of male infertility is increased levels of seminal reactive oxygen species (ROS). ROS plays a crucial role in several reproductive steps – in normal development and maturation of spermatozoa, capacitation, acrosome reaction and fertilization [4,5]. Endogenous sources of ROS include leukocytes and immature sperm cells in semen and mitochondria in spermatozoa [6,7]. Exogenous sources include inflammatory reactions and diseases of the male genital tract [8]. Excessive levels of ROS can damage normal spermatozoa by inducing lipid peroxidation and DNA damage [9-12] and are associated with poor sperm function and subfertility [5,13-17]. An abnormal increase in ROS levels can overwhelm local antioxidants and lead to oxidative stress [18]. High ROS levels in semen [19] have been found in 25-40% of infertile men and in 40-88% of subfertile patients [20]. Seminal oxidative stress can be detected by measuring ROS concentrations [21,22]. ROS levels can be easily measured with a chemiluminescence assay [22-24]. In evaluating the cause of male infertility, semen analysis usually fails to provide an answer [2]. Thus, there is an urgent need for robust markers that may help in the assessment of sperm function or its fertilizing capacity. Measurement of seminal ROS levels has become important as ROS levels are significantly correlated with the fertilization rate in infertile couples undergoing IVF [25]. Although there is no single standardized method for measuring pathological value of ROS levels in infertile men, chemiluminescence is a common method. We have previously reported that higher levels of ROS can be considered an independent marker of male infertility, one that is not dependent on normal or abnormal semen parameters [26]. This information could be crucial in the inclusion of ROS measurement in routine diagnostic examination for idiopathic male infertility.

A negative correlation between ROS production and percentage of normal sperm, concentration, and motility has been previously demonstrated [27,28]. We have also reported similar results in a fertile population [27]. However, the correlation between ROS and semen parameters among proven fertile donors who have fathered a child within the past 2 years and infertile patients has not been studied. Therefore, we sought to study the correlation between semen parameters and ROS levels amongst different groups of fertile donors.

The purpose of our study was to 1) determine the relationship between ROS levels and traditional semen parameters in fertile donors and infertile patients, and 2) establish reference ROS values of levels in normal healthy donors of proven and unproven fertility compared with an infertile group of men.

## Methods

### Sample collection and preparation

The Cleveland Clinic Institutional Review Board approved this study. IRB consent approved by the Cleveland Clinic was provided to each subject, and the purpose of the study was clearly explained. If the participant was interested, a written signature was obtained and witnessed before he was enrolled in the study. Semen samples were collected from men with a history of infertility (All Patients; Group 1; n = 318) who came to Cleveland Clinic for infertility treatment and normal, healthy men. Infertile men were referred for routine semen analysis and measurement of oxidative stress markers. The inclusion criteria were: 1) all subjects were attending the male infertility clinic for fertility issues, 2) all of these men were evaluated for proven male-factor infertility as assessed the male infertility specialist, 3) all of them underwent history, physical and laboratory evaluation and 4) all female partners of the infertile men had undergone gynecologic evaluation and had normal results on a fertility workup. The exclusion criteria were: history of smoking, illicit drug use; exposure to any environmental or occupational toxicants; use of medication with proven toxicity on fertility; exposure to radiation or heat; orchitis due to mumps; sexually transmitted or systemic diseases; cryptorchidism; testicular torsion; genitourinary anomalies; epididymal or vas deferens alterations; and inguinal surgery. In addition, subjects were not included if they presented with azoospermia, cryptorchidism, incomplete semen analysis results or inadequate semen sample for measurement of ROS.

The control group comprised of 56 healthy men. All subjects were asked to fill out a brief questionnaire about their lifestyle, past illness, sexual behavior, smoking, use of alcohol and recreational drugs and if they had initiated a pregnancy in the past. Controls were divided into three groups:

Group 2: All Donors (n = 56). In this group, 44 subjects had initiated a pregnancy and 12 had not. The inclusion criteria were: 1) normal semen parameters; 2) no sexually transmitted infections; 3) no recreational drug use 4) may or may not have initiated a pregnancy in the past. The exclusion criteria were: azoospermia, incomplete semen analysis results or inadequate semen sample for measurement of ROS.

Group 3: Proven Donors (n = 28). The inclusion criteria were: 1) normal semen parameters; 2) no sexually transmitted infections 3) no recreational drug use and 4) must have initiated a pregnancy in the past. The exclusion criteria were the same as for the All Donors group.

Group 4: Proven Donors who initiated a recent pregnancy i.e. within the last 2 years (<2 years) (n=16). The inclusion criteria were: 1) normal semen parameters 2) sexually transmitted infections 3) no recreational drug use and 4) must have initiated a pregnancy within the last 2 years. The exclusion criteria were similar to the above 2 donor groups.

### Semen analysis

After complete liquefaction at 37°C for 20 minutes, 5 μL of each specimen was loaded on a 20 μL MicroCell chamber (Vitrolife, San Diego, CA) and analyzed for sperm concentration and motility according to World Health Organization (WHO) guidelines [29]. Viability was determined using Eosin - Nigrosin stain. A minimum of 200 spermatozoa were counted per sample. Seminal smears were stained with Diff-Quik stain (Baxter Healthcare, McGaw Park, IL), and normal sperm morphology was assessed according to the WHO 2010 criteria [29].

### Measurement of white blood cells

Samples with a high concentration of round cells (>5 per high power field) were examined for the presence of white blood cells especially the granulocytes by the peroxidase or the Endtz test [24]. To conduct the Endtz test, a 20 μL well-mixed aliquot of the semen sample was mixed with one volume of PBS and 2 volumes of working Endtz solution in an amber-colored eppendorf tube. After 5 minutes, a drop of the aliquot was placed on a Makler chamber and examined for the presence of dark brown cells under a ×10 bright field objective. Leukocytospermia was defined as the presence of >1 × 10^6^ WBC/mL according to the WHO criteria [29].

### Measurement of reactive oxygen species

After complete liquefaction, ROS levels were measured with a chemiluminescence assay using luminol as the probe (5-amino2,3-dihydro-1,4-phthalazinedione; Sigma Chemical Co., St. Louis, MO). A 100 mmoL/L stock solution of luminol was prepared in dimethyl sulfoxide. For the analysis, 10 μL of the working solution (5 mmol) was added to 400 μL of neat sperm sample. Chemiluminescence was measured for 15 minutes with an Autolumat LB 953 luminometer (AutoLumat Plus LB 953, Oakridge, TN) in the integrated mode. Results were expressed as relative light units/sec (RLU/s) [30].

### Statistical analysis

Donor and patient groups were compared with respect to quantitative parameters, including semen parameters and ROS, using Wilcoxon rank sum tests. Associations between quantitative measures (sperm concentration, motility, and morphology) were assessed with Spearman correlation coefficients. The distribution of quantitative parameters was described among infertile patients and fertile donors using mean ± standard deviation, with ROS levels also described using median (25th and 75th percentile). A receiver operating characteristic curve (ROC) was used to assess the ability of ROS as a means of distinguishing patient and fertile donor values. A cutoff value was chosen that maximized the sum of estimated sensitivity and specificity. All analyses were performed with use of R version 2.11.1 (The R Foundation, http://www.R-project.org). P values of <0.05 were considered statistically significant.

## Results

### Semen parameters across donors and patients

**Figure 1:**
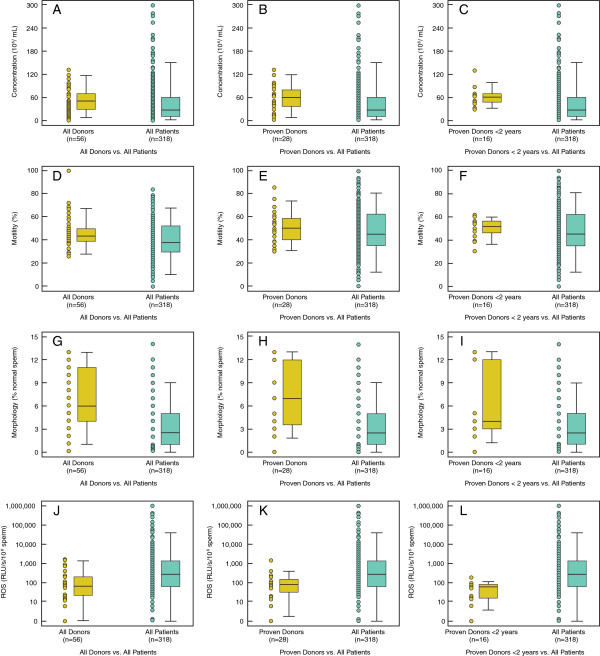
**Box Plots for semen parameters of Donors vs. Infertile Patients.** Box plots comparing between All Donors, Proven Donors and Proven Donors <2 years and All Patients for concentration **(A-C)**; motility **(D-F)** and morphology **(G-I)** and ROS levels in **J**: All Donors and All Patients; **K**: Proven Donors and All Patients; **L**: Proven Donors <2 years and All Patients. The box plots show the width and the whiskers. The width of the box is proportional to the size of the group. The bottom and the top of the box represent the 25th and 75^th^ percentile. The band in the box is the median. The whiskers represent the standard deviation. These box plots show that concentration, motility, and morphology vary between 3 groups of donors and patients

The distribution of semen parameters for All Patients (group 1) and All Donors (group 2) are shown in Table 1. Concentration, % motility, and normal morphology were higher in All Patients (group 1) versus All Donors (group 2) and also when Proven Donors (group 3) and Proven Donors < 2 years (group 4) were compared with All Patients (group 1). Figure 1A-I are box plots showing the differences for concentration (A-C), motility (D-F) and morphology (G-I) in groups 2, 3, and 4 compared to All Patients (group 1).

**Table 1:** Semen parameters in all donors, proven donors and proven donors <2 years compared with patients

**Parameter**	**Donors**	**Patients**	**P value**
**All donors vs. all patients**			
**Volume (mL)**	3.36 ± 2.02	3.15 ± 1.49	0.95
**Concentration (X10**^**6**^**/mL)**	54.26 ± 32.19	45.33 ± 51.00	**0.001**
**Motility (%)**	53.70 ± 15.00	46.95 ± 20.16	**0.016**
**Endtz(X10**^**6**^**/mL)**	1.04 ± 2.54	0.50 ± 1.62	0.41
**Normal morphology (%)**	6.93 ± 3.91	3.20 ± 2.91	**<0.001**
**ROS (RLU/s)**	228.80 ± 396.27	12540.40 ± 70846.85	**<0.001**
	64.8(21.1,198.2)^a^	267.5(59.4, 1320)^a^	
**Proven donors vs. all patients**			
**Volume (mL)**	4.24 ± 2.13	3.15 ± 1.49	**0.008**
**Concentration (X10**^**6**^**/mL)**	60.07 ± 33.44	45.33 ± 51.00	**0.002**
**Motility (%)**	50.85 ± 13.52	46.95 ± 20.16	0.30
**WBC X10**^**6**^**/mL)**	0.00 ± 0.00	0.50 ± 1.62	0.09
**Normal morphology (%)**	7.00 ± 4.35	3.20 ± 2.91	**<0.001**
**ROS (RLU/s)**	149.46 ± 275.08	12540.40 ± 70846.85	**<0.001**
	75.8(33.3, 147.8)^a^	267.5(59.4, 1320)^a^	
**Proven donors <2y vs. all patients**			
**Volume (mL)**	5.03 ± 2.22	3.15 ± 1.49	**<0.001**
**Concentration (X10**^**6**^**/mL)**	61.59 ± 23.93	45.33 ± 51.00	**0.002**
**Motility (%)**	49.88 ± 8.68	46.95 ± 20.16	0.40
**Endtz(X10**^**6**^**/mL)**	0.00 ± 0.00	0.50 ± 1.62	0.18
**Normal morphology (%)**	6.77 ± 4.95	3.20 ± 2.91	**0.006**
**ROS (RLU/s)**	54.53 ± 45.31	12540.40 ± 70846.85	**<0.001**
	75.8(33.3, 147.8)^a^	267.5(59.4, 1320)^a^	

ROS = reactive oxygen species; RLU = relative light units; Values are mean ± SD. WBC = white blood cells measured by the Endtz test.

^a^median (25^th^, 75^th^ percentile); P < 0.05 considered significant by Wilcoxon rank sum test.

### Correlations with semen parameters

In All Donors (group 2), morphology significantly correlated with concentration (r = 0.41; P = 0.009) and motility (r = 0.51; P < 0.001). In Proven Donors (group 3), morphology was related with concentration (r = 0.48; P = 0.037) and motility (r = 0 49; P < 0.032). In Proven Donors <2 years (group 4), only morphology was correlated with concentration (r = 0.73, P = 0.004).

Among All Patients (group 1), sperm concentration was related with motility (r = 0.54; P < 0.001), and normal morphology (r = 0.38; P < 0.001); motility was correlated with normal morphology (r = 0.47; P < 0.001). Similarly, when All Donors (group 2) was compared with All Patients (group 1), sperm concentration was significantly correlated with motility (r = 0.51; P < 0.001) and normal morphology (r = 0.37; P < 0.001); motility and normal morphology (r = 40; P < 0.001). In Proven Donors <2 years (group 4) and All Patients (group 1), motility was correlated with concentration (r = 0.54; P < 0.001) and normal morphology (r = 0.37; P < 0.001); and concentration was correlated with morphology (r = 0.35; P < 0.001).

**Figure 2:**
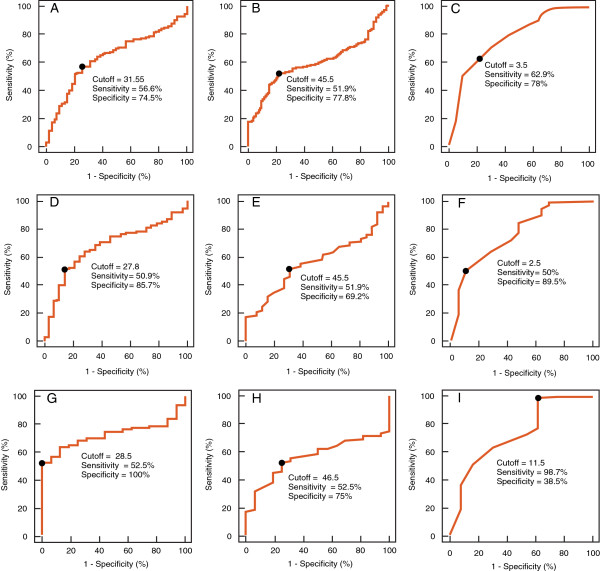
**Receiver operating characteristic curves showing cutoff value, sensitivity and specificity for All Donors and All Patients for A: concentration (AUC = 0.640); B: motility (AUC = 0.602) and C: morphology (AUC = 0.775); Proven Donors and All Patients for D: concentration (AUC = 0.674); E: motility (AUC = 0.562) and F: morphology (AUC = 0.765) and All Proven Donors <2 years established pregnancy in the last 2 years and All Patients. G**: concentration (AUC = 0.728); **H:** motility (AUC = 0.562) and **I**: morphology (AUC = 0.723).

We also examined the cutoff values, sensitivity and specificity and area under the curve from the receiver operating characteristic curve for concentration, motility and morphology for donors and patients (Table 2). A cutoff value of 31.55 X10^6^/mL for concentration, 45.5% for motility and 3.5% for normal morphology provided a specificity of 74.5%, 77.8% and 78.0%, respectively. The ROC curves for the 3 groups compared with All Patients for concentration, motility and morphology are shown in Figure 2A-I.

**Table 2:** Receiver operator characteristic curve analysis for concentration, motility and normal morphology for 3 donor groups and infertile men

**Parameter**	**Group**	**Cut-off**	**Sensitivity**	**Specificity**	**AUC**
	**All donors + All patients**				
**Concentration (X10**^**6**^**/mL)**		**31.55**	**56.6**	**74.5**	**0.640**
**Motility (%)**		**45.5**	**51.9**	**77.8**	**0.602**
**Morphology (%)**		**3.5**	**62.9**	**78.0**	**0.775**
	**Proven donors + All patients**				
**Concentration (X10**^**6**^**/mL)**		**27.8**	**50.9**	**85.7**	**0.674**
**Motility (%)**		**45.5**	**51.9**	**69.2**	**0.562**
**Morphology (%)**		**2.5**	**50.0**	**89.5**	**0.765**
	**Proven donors < 2 years + All patients**				
**Concentration (X10**^**6**^**/mL)**		**28.5**	**52.5**	**100**	**0.728**
**Motility (%)**		**46.5**	**52.5**	**75.0**	**0.562**
**Morphology (%)**		**11.5**	**98.7**	**38.5**	**0.723**

### ROS and semen parameters

**Figure 3:**
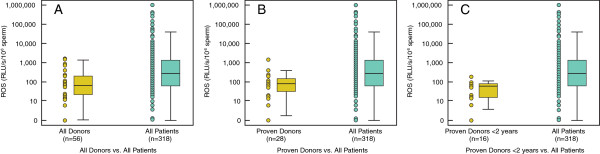
**Receiver operating characteristic curves for ROS showing cutoff value, sensitivity and specificity. A**: All Donors and All Patients; **B**: Proven Donors and All Patients; **C**: Proven Donors <2 years of established pregnancy and All Patients. The area under curve for All Donors vs. All Patients was 0.683; Proven Donors vs. All Patients was 0.783 and Proven Donors <2 years of established pregnancy and All Patients was 0.785.

Significantly higher levels of ROS [median (25th, 75^th^ percentile) RLU/s)] were seen in the All Patients (group 1) compared to All Donors (group 2); 267.5(59.4, 1320) vs. 64.8(21.1, 198.2) (P < 0.001); Proven Donors (group 3); 267.5(59.4, 1320) vs. All Patients (group 1) 75.8(33.3, 147.8) (P < 0.001) and Proven Donors <2 years (group 4); 267.5(59.4, 1320) vs. All Patients (group 1) 75.8(33.3, 147.8) (P < 0.001) (Table 1). Figure 1J-L shows the box plots for the ROS levels in the 3 donor groups compared with All Patients. In All Donors (group 2) and Proven Donors (group 3): ROS was negatively associated with sperm concentration (r = −0.0351 (P = 0.021 and r = −0.377; P = 0.05), respectively. When ROS levels were examined in All Donors (group 2) and All Patients (group 1): ROS was negatively correlated with ejaculate volume (r = −0.111; P = 0.046), concentration (r = −0.373; P < 0.001) and motility (r = −0.265; P < 0.001). Similarly, ROS was negatively correlated with volume, concentration and motility in Proven Donors (group 3) and Proven Donors <2 years (group 4) compared with All Patients (group 1). We also examined the cutoff values, sensitivity and specificity from the receiver operating characteristic curve for ROS in All Patients (group 1) and donor groups 2–4 (All Donors, Proven Donors and Proven Donors <2 years) (Figure 3A-C). The area under curve for All Donors vs. All Patients was 0.683; Proven Donors vs. All Patients was 0.783 and Proven Donors <2 years and All Patients was 0.785.

## Discussion

The purpose of our study was to 1) determine the relationship between ROS levels and traditional semen parameters in fertile donors and infertile patients, and 2) establish reference values for ROS levels in normal healthy donors of proven and unproven fertility compared with an infertile group of men. The first part of the study showed that ROS was negatively correlated with sperm concentration, motility, and sperm morphology. Similar correlations were reported in other studies [13,27,28,31,32]. On the other hand, some studies showed no significant relationship between sperm motility and the levels of ROS production [33]. Pasqualotto et al. also found similar findings to those of Whittington et al. on association between seminal oxidative stress in patients presenting with prostatitis and semen parameters [17]. In both of these studies, washed semen samples were utilized in the analysis in which white blood cells were excluded from the semen such as in cases of leukocytospermia.

In the second part of the study, we found that semen parameters were correlated with ROS in different donor groups, thus making our results more applicable to infertility management. Our study results are similar to other studies in that the sperm concentration, motility, and normal morphology were significantly lower in the infertile men with higher levels of ROS [27,31,32]. Monitoring ROS levels in fertile men and more so in fertile men with proven fertility in the past 2 years is important to rule out high ROS levels in these men before any clinical association of ROS can be made in the evaluation of infertile patients. Hence, studying the correlation between ROS levels and sperm parameters in different donor groups becomes extremely crucial for evaluating male infertility.

The pathologic effects of ROS on the male reproductive system have been demonstrated by several studies [34,35]. But there are limited studies correlating seminal ROS levels to pregnancy outcomes [16,36-38] due to the lack of proper controls. However, defining a standardized cutoff value for pathologic levels of ROS is important. The problem exists due to the lack of agreement on a universal method for ROS measurement, making comparison between the studies difficult. Subsequently, there is a lack of randomized controlled trials and additionally a lack of standardized therapies for the treatment of elevated ROS levels [39]. We were interested in providing a cutoff value for ROS that could be considered pathologic by using our established methodology [30].

In the present study, receiver operating characteristic curve analysis was performed to find a cutoff value that could be used for diagnostic purposes in case of male factor infertility. We suggest a cutoff of 91.95 RLUs to be used 1) as a diagnostic or screening tool in general (to diagnose male factor infertility), 2) as a prognostic tool in assisted reproduction, or 3) for therapeutic interventions. Reactive oxygen species–positive values can diagnose male factor infertility with a sensitivity of 68.8% and 93.8% specificity. We have also established cutoff values for sperm concentration at 31.55, 45.5% for motility and 3.5% for normal morphology with specificity of 74.5%, 77.8% and 78%, respectively. These results are consistent with previously established cutoff values for sperm parameters in fertile and subfertile groups [40].

Seminal oxidative stress measurement is also important as a predictive tool in assisted reproductive technology clinics [41,42]. Measuring ROS levels prior to beginning assisted reproductive techniques will help identify the cause of high ROS generation, rule out leukocytospermia and suggest antibiotics if infection is suspected [43,44]. Use of oral antioxidant supplements and addition of antioxidants in sperm preparation media and assisted reproductive technology media may lead to positive pregnancy outcome [26,45-47]. Our study suggests that a ROS level of 91.95 RLUs should be considered physiologic and infertility patients with a level higher than this cutoff should be considered for antioxidant supplementation. The high sensitivity, specificity and area under the curve for All Donors vs. All Patients, Proven Donors vs. All Patients and Proven Donors <2 years of established pregnancy and All Patients suggests the usefulness of ROS as an additional tool in screening infertile patients.

One of the strengths of this study is the large number of participants, which improved the efficacy of identifying the cutoff value of normal ROS levels in the population. We also included healthy donors with proven fertility. Most studies demonstrating normal reference values for ROS have used healthy donors with unknown fertility status as controls. One of the reasons for this is the difficulty in recruiting a fertile population. However, healthy donors presenting with normal semen parameters cannot be considered good controls until their fertility potential is fully evaluated. A limitation in this study was that we did not categorize the infertile men based on their clinical diagnoses.

## Conclusions

In conclusion, high levels of ROS in semen may be a causative factor for male infertility in patients. Determining ROS levels thus forms an important measure of the diagnostic work up of patients with idiopathic infertility. A review of the current literature reveals an inconsistent effect of therapies aimed at reducing seminal ROS upon clinical outcomes. We suggest a need for inclusion of ROS testing in patients with idiopathic infertility. This prospective study helps define a reference range for ROS levels in semen. This will aid in the appropriate diagnosis and treatment of patients with oxidative stress and thereby improve sperm quality and fertility.

## Competing interests

Authors declare that they have no competing interests.

## Authors’ contributions

AA conceived the idea, supervised the study, and edited the article for submission. SA and DD conducted the study and helped with the data collection and management of this study. RS assisted with data analysis, writing of manuscript, and preparation for submission. MA, AMA, RKS and ES helped with the reviewing and editing of the manuscript. All authors read and approved the final manuscript.
